# Predictors of Problematic Internet Use Among Gifted Children: The Role of Health Literacy and Health-Promoting Lifestyle Beliefs

**DOI:** 10.3390/children13081110

**Published:** 2026-08-19

**Authors:** Emine Zahide Özdemir, Murat Bektaş, Necati Özcan, Beren Çağla Erengönül

**Affiliations:** 1 Department of Pediatric Nursing, Faculty of Nursing, Dokuz Eylül University, 35340 İzmir, Türkiye; murat.bektas@deu.edu.tr; 2 Konak Şehit Ömer Halisdemir Science and Art Center, Ministry of National Education, 35280 İzmir, Türkiye; turkdilibilimi@gmail.com (N.Ö.); berenerengonul@gmail.com (B.Ç.E.)

**Keywords:** gifted children, problematic internet use, health literacy, health-promoting lifestyle beliefs, school health, self-regulation

## Abstract

**Highlights:**

**What are the main findings?**

- After adjustment for sociodemographic and lifestyle covariates, health beliefs—not physical activity or nutrition beliefs—were significantly and negatively associated with problematic internet use (PIU) among gifted school-aged children.
- Health literacy domains did not add statistically significant incremental variance in explaining PIU beyond covariates and health-promoting lifestyle beliefs.

**What are the implications of the main findings?**

- School health interventions for gifted children may benefit from targeting internalized health beliefs in addition to information-based health education alone.
- Belief-focused, tailored programs could inform more equitable school health services for gifted students; longitudinal research is needed to establish directionality.

**Abstract:**

**Background/Objectives:** Problematic internet use (PIU) is a growing behavioral health concern among school-aged children. Whether health literacy and health-promoting lifestyle beliefs are associated with PIU among gifted children—a developmentally distinct population—has, to the best of our knowledge, not been investigated. **Methods:** A cross-sectional correlational design was used with 118 gifted students (M = 13.80, SD = 2.06) attending a Science and Art Center (BİLSEM) in İzmir, Türkiye. Data were collected using the Problematic Internet Use Scale–Adolescent Form (PIUS-A; α = 0.92), the Health Literacy for School-Aged Children Scale (HLSAC; α = 0.87), and the Adolescent Health-Promoting Lifestyle Beliefs Scale (AHPLBS; α = 0.91) in a classroom setting under researcher supervision. A three-step hierarchical multiple linear regression was performed, with age, sex, daily sleep duration, daily screen time, and weekly physical activity entered as covariates in the first step. **Results:** Covariates explained 24.8% of the variance in PIU (*p* < 0.001), with daily screen time as the strongest predictor (β = 0.36). Health-promoting lifestyle beliefs added significant incremental variance (ΔR^2^ = 0.063, *p* = 0.023); health beliefs were the only statistically significant belief predictor (β = −0.24, *p* = 0.036), with lower health belief scores associated with higher PIU. Health literacy domains did not add statistically significant incremental variance (ΔR^2^ = 0.028, *p* = 0.495), and no individual domain was a statistically significant predictor. **Conclusions:** Within the constraints of a cross-sectional design, health beliefs appear to play a more prominent role than health literacy in the covariate-adjusted explanation of PIU among gifted school-aged children. School health nurses may prioritize interventions that strengthen internalized health beliefs alongside information-based education, contributing to more equitable, tailored school health services for this population.

## 1. Introduction

The widespread integration of digital technologies into the daily lives of school-aged children has raised growing concerns about problematic internet use (PIU), characterized by compulsive online behaviors, diminished self-control, and interference with daily functioning [1,2,3]. PIU has been associated with a range of adverse health outcomes in children and adolescents, including disrupted sleep, reduced physical activity, emotional dysregulation, poorer mental health, and impaired academic performance [4,5,6,7,8]. Given these consequences, PIU represents a significant behavioral health concern for school communities, warranting proactive identification and prevention efforts by school health professionals [9].

Prior research has examined several health-related factors associated with PIU in general adolescent populations. Health literacy—defined as the capacity to access, understand, evaluate, and apply health information—has been proposed as a protective factor against risk-related behaviors, including excessive internet use [10,11,12,13,14]. Similarly, health-promoting lifestyle beliefs, encompassing positive beliefs about physical activity, nutrition, and personal health responsibility, have been linked to self-regulatory behaviors that may buffer against maladaptive digital habits [15,16]. However, existing studies have predominantly examined these relationships in general student populations, with limited attention to subgroups whose developmental characteristics may fundamentally alter how health literacy and lifestyle beliefs relate to digital behavior [17,18]. Critically, whether health literacy domains and health-promoting lifestyle beliefs are associated with PIU among gifted school-aged children—a population with distinct cognitive and socio-emotional profiles—has, to the best of our knowledge, not been investigated; a structured search of PubMed, Scopus, and Web of Science (August 2026) identified no study jointly examining these factors in a gifted pediatric sample.

Gifted children constitute a developmentally distinct subgroup whose relationship with digital technology warrants specific investigation. Their heightened intellectual curiosity and advanced cognitive abilities may intensify engagement with online environments as sources of exploration and stimulation beyond what standard curricula offer [19,20,21]. At the same time, research challenges the assumption that cognitive ability confers behavioral resilience: gifted children are reported to experience heightened emotional sensitivity, challenges with self-regulation, and difficulties maintaining behavioral balance—characteristics that may increase susceptibility to unregulated internet use independent of cognitive competence [22,23,24]. Consequently, applying findings from general adolescent populations to gifted children may be inappropriate. In particular, the role of health literacy may differ in this population: gifted children typically demonstrate advanced information-processing skills and high health knowledge levels, potentially reducing variability in health literacy scores and diminishing its predictive value for behavioral outcomes [25]. By contrast, internalized health beliefs and lifestyle orientations—which reflect motivational and value-based dimensions of health behavior rather than cognitive competence alone—may represent more relevant correlates of PIU in this group.

School health nurses and other school health professionals are well positioned to implement early, targeted interventions to prevent PIU among high-risk student populations [9]. However, evidence identifying which specific health-related factors are associated with PIU among gifted children remains limited, leaving school health practice without a clear empirical basis for tailored intervention design [17,26]. Therefore, this study aimed to examine whether health literacy domains and health-promoting lifestyle beliefs are associated with PIU among gifted school-aged children attending a specialized educational center in Türkiye, after adjustment for sociodemographic and lifestyle characteristics. Specifically, the following research questions were addressed: (1) Do health-promoting lifestyle belief subdimensions—health beliefs, physical activity beliefs, and nutrition beliefs—explain variance in PIU among gifted school-aged children beyond sociodemographic and lifestyle covariates? (2) Do health literacy domains—theoretical knowledge, practical knowledge, critical thinking, self-awareness, and citizenship—explain additional variance in PIU beyond these covariates and lifestyle beliefs?

## 2. Materials and Methods

### 2.1. Participants

The study was conducted at Konak Şehit Ömer Halisdemir Science and Art Center (BİLSEM) in İzmir, Türkiye. Science and Art Centers (BİLSEM) are public institutions affiliated with the Turkish Ministry of National Education, where students are identified as gifted through a standardized, multi-stage national assessment process. A convenience sample was used, consisting of school-aged students attending the center who had been officially identified as gifted by the Turkish Ministry of National Education. Participants were aged 10–15 years (M = 13.80, SD = 2.06).

Sample size was determined a priori using G*Power 3.1 software based on the correlation (r = 0.416) reported by [27] between e-health literacy and health-promoting behaviors among adolescents. This correlation corresponds to R^2^ = 0.173 and an effect size of f^2^ = 0.21, a medium effect according to Cohen [28]. With α = 0.05, statistical power of 0.95, and five predictors (the largest predictor set defined by the research questions at the design stage), the minimum required sample size was 101 participants. A total of 118 students were enrolled, exceeding this requirement. The final covariate-adjusted model including thirteen predictors was specified during the revision process and was not part of the a priori calculation; this is acknowledged in the limitations.

Inclusion criteria were: (1) official identification as gifted by the Turkish Ministry of National Education, (2) active enrollment at the BİLSEM center during the data collection period, (3) voluntary participation with written parental informed consent and child assent, and (4) sufficient Turkish language proficiency to complete the questionnaires. Students who did not provide consent or withdrew during data collection were excluded.

### 2.2. Measures

**Sociodemographic Information Form.** A researcher-developed form collected data on participants’ age, sex, height, weight, daily sleep duration, daily screen time, weekly physical activity frequency, and presence of chronic disease. Age, sex, daily sleep duration, daily screen time, and weekly physical activity were included as covariates in the regression analysis. *Daily sleep duration, daily screen time, and weekly physical activity were recorded using ordered response categories rather than exact continuous values.*

**Problematic Internet Use Scale–Adolescent Form (PIUS-A).** The PIUS-A was originally developed by Ceyhan, Ceyhan, and Gürcan (2007) and adapted for adolescents by Ceyhan and Ceyhan (2014) [29]. This 27-item, 5-point Likert-type scale (1 = not appropriate at all to 5 = completely appropriate) assesses PIU across three dimensions: Negative Consequences of the Internet, Excessive Use, and Social Benefit/Social Comfort. Example items include “Whenever I decide to disconnect from the internet, I say to myself, ‘just a few more minutes’” (item 1) and “I neglect my daily tasks in order to spend more time on the internet” (item 14). Higher scores indicate higher levels of problematic use. In the original adaptation study, Cronbach’s α was 0.93 [29]; in the present study, α for the total scale—the only PIUS-A score used in the analyses—was 0.92. Following the published scoring procedure, only items 7 and 10 were reverse-coded before the 27 items were summed; the Social Benefit/Social Comfort dimension was not reverse-coded as a whole [29]. Thus, higher total scores consistently indicate greater problematic internet use.

**Health Literacy for School-Aged Children Scale (HLSAC).** Developed by Paakkari et al. and adapted into Turkish by Haney (2018) [30], this 10-item, 4-point Likert scale (1 = not true at all to 4 = absolutely true) evaluates five health literacy domains, each measured by two items: theoretical knowledge, practical knowledge, critical thinking, self-awareness, and citizenship. All items follow the stem “I am confident that…”; example items include “…I have good knowledge about health” (item 1, theoretical knowledge) and “…when necessary, I can find health-related information that is easy for me to understand” (item 7, practical knowledge). The Turkish adaptation demonstrated strong construct validity (RMSEA = 0.035, CFI = 0.99, GFI = 0.99; α = 0.77) [30 In the Turkish adaptation, α = 0.90]. In the present study, α for the total scale was 0.87. Because the five two-item domain scores served as predictors, domain-level internal consistency was also computed: α = 0.61 (inter-item r = 0.44) for theoretical knowledge, α = 0.56 (r = 0.40) for practical knowledge, α = 0.54 (r = 0.38) for critical thinking, α = 0.63 (r = 0.46) for self-awareness, and α = 0.47 (r = 0.31) for citizenship. These modest values, expected for two-item indices, are addressed in the limitations.

**Adolescent Health-Promoting Lifestyle Beliefs Scale (AHPLBS).** Originally developed by Melnyk et al. and adapted into Turkish by Akdeniz Kudubeş and Bektaş (2020) [31], this 16-item, 5-point Likert scale (1 = strongly disagree to 5 = strongly agree) measures health-promoting lifestyle beliefs across three subscales: Health Beliefs (7 items), Physical Activity (5 items), and Nutrition (4 items). Example items include “I believe that I can reach the goals I set for myself” (item 5, health beliefs) and “I believe that I can be more active” (item 7, physical activity). In the Turkish adaptation, α = 0.90 [31]; in the present study, α was 0.91 for the total scale, 0.87 for Health Beliefs, 0.77 for Physical Activity, and 0.85 for Nutrition.

### 2.3. Procedure

Data were collected between December 2025 and January 2026. Following ethical approval and institutional permission, parental informed consent forms were distributed to eligible students in advance. Students who returned signed parental consent forms completed the questionnaires in a classroom setting at the BİLSEM center under researcher supervision. Prior to completion, students were informed that there were no right or wrong answers and that they should select the response that best reflected their own experiences. Completion of the questionnaires took approximately 20–25 min. Participation was entirely voluntary and anonymous, and no incentives were provided. All returned questionnaires were checked for completeness prior to data entry.

### 2.4. Data Analysis

Data were analyzed using IBM SPSS Statistics 25.0. Descriptive statistics were used to summarize participant characteristics and scale scores. Pearson correlation coefficients were computed among the PIU total score, HLSAC domains, and AHPLBS subscales; the sociodemographic and lifestyle covariates were not included in this correlation matrix. Age was entered as a continuous variable in years, and sex was dummy-coded (0 = female, 1 = male). Daily sleep duration (<6, 7–8, 9–10, and ≥11 h), daily screen time (<2, 3–4, 5–6, and >7 h), and weekly physical activity (none, 1–2, 3–4, and ≥5 days/week) were entered as ordinal scores coded 1–4 in the listed order. Accordingly, the unstandardized coefficient for each ordinal covariate represents the expected change in PIU total score associated with a one-category increase. To address the research questions while controlling for sociodemographic and lifestyle characteristics previously associated with PIU [32,33,34], a three-step hierarchical multiple linear regression was conducted with the PIU total score as the dependent variable. Age, sex, daily sleep duration, daily screen time, and weekly physical activity were entered in Step 1 as covariates; the three AHPLBS subscales (health beliefs, physical activity beliefs, nutrition beliefs) were entered in Step 2 (Research Question 1); and the five HLSAC domains were entered in Step 3 (Research Question 2). This single hierarchical model replaced the two separate regression analyses reported in the original submission, allowing the incremental contribution of each predictor block to be estimated while controlling for the covariates and the other block. Regression assumptions were formally examined: linearity and homoscedasticity were evaluated through plots of standardized residuals against standardized predicted values, normality of residuals through the histogram and normal P–P plot, independence of residuals through the Durbin–Watson statistic (2.05), and multicollinearity through tolerance and variance inflation factors (all tolerance ≥ 0.33; all VIF ≤ 3.02). Statistical significance was set at *p* < 0.05 (two-tailed).

## 3. Results

### 3.1. Participant Characteristics

Data from 118 gifted school-aged children were included in the analysis. The mean age of participants was 13.80 ± 2.06 years, and slightly more than half were female (55.1%). Most participants did not report any chronic disease (85.6%). Regarding lifestyle characteristics, the majority reported sleeping 7–8 h per night (65.3%) and spending 3–4 h per day on screen-based activities (45.8%). Approximately one-third reported no regular weekly physical activity (28.8%). Demographic and lifestyle characteristics are presented in Table 1; descriptive statistics and internal-consistency coefficients for the study measures are presented in Table 2.

### 3.2. Bivariate Correlations

PIU total scores showed significant negative bivariate correlations with health beliefs (r = −0.40, *p* < 0.001), nutrition beliefs (r = −0.38, *p* < 0.001), self-awareness (r = −0.27, *p* = 0.004), citizenship (r = −0.23, *p* = 0.011), physical activity beliefs (r = −0.22, *p* = 0.017), and practical knowledge (r = −0.21, *p* = 0.020). Correlations of PIU with theoretical knowledge (r = −0.16, *p* = 0.083) and critical thinking (r = −0.13, *p* = 0.151) were not statistically significant. Intercorrelations among predictors ranged from r = 0.14 to r = 0.68 (Table 3).

### 3.3. Hierarchical Regression Analysis

In Step 1, the sociodemographic and lifestyle covariates explained 24.8% of the variance in PIU (R^2^ = 0.248, adjusted R^2^ = 0.214, F(5,112) = 7.37, *p* < 0.001); daily screen time (β = 0.36, *p* < 0.001) and weekly physical activity (β = −0.19, *p* = 0.031) were statistically significant. In Step 2, adding the three health-promoting lifestyle belief subscales significantly improved the model (ΔR^2^ = 0.063, F change(3,109) = 3.32, *p* = 0.023; R^2^ = 0.311). Health beliefs were the only statistically significant belief predictor (B = −0.79, SE = 0.37, β = −0.24, *p* = 0.036); lower health belief scores were associated with higher PIU scores. Physical activity beliefs (β = 0.05, *p* = 0.617) and nutrition beliefs (β = −0.09, *p* = 0.472) were not statistically significant. In Step 3, the five health literacy domains did not add statistically significant incremental variance (ΔR^2^ = 0.028, F change(5,104) = 0.88, *p* = 0.495; R^2^ = 0.339), and none of the individual domains was a statistically significant predictor. In the full model, daily screen time remained the strongest predictor (β = 0.29, *p* = 0.002). Full coefficients are presented in Table 4.

## 4. Discussion

This study examined whether health literacy domains and health-promoting lifestyle beliefs are associated with PIU among gifted school-aged children after adjustment for sociodemographic and lifestyle characteristics. Lifestyle beliefs—particularly health beliefs—added significant incremental variance in explaining PIU beyond the covariates, whereas health literacy domains did not. These findings suggest that motivational and value-based dimensions of health, rather than knowledge-based competencies alone, may be more proximally associated with problematic internet behaviors among gifted children; given the cross-sectional design, however, no causal ordering can be inferred.

### 4.1. The Role of Health Beliefs in Problematic Internet Use

In the present study, lower health belief scores were associated with higher PIU scores even after adjustment for age, sex, sleep duration, screen time, and physical activity, indicating that gifted children who place less value on personal health responsibility and self-care may be more vulnerable to poorly regulated internet use. This finding is consistent with prior research demonstrating that health beliefs are associated with risk-related behaviors and self-regulatory capacity in children and adolescents [15,16,35,36]. From a theoretical perspective—and as an interpretive hypothesis that was not directly tested in this study—strong internalized health beliefs may support the establishment of personal limits, facilitate goal-directed behavior, and enable children to balance digital activities with other daily routines [37,38,39]. Notably, among gifted children, advanced cognitive capacity does not appear to translate automatically into effective behavioral self-regulation—a pattern consistent with the broader literature on socio-emotional development in this population [22,23,24]. These findings suggest that health beliefs may function as a particularly relevant correlate and potential protective factor against PIU in gifted children, operating through motivational and value-based pathways rather than through cognitive competence alone; longitudinal and experimental designs are required to test this proposition.

### 4.2. Physical Activity and Nutrition Beliefs

Physical activity beliefs and nutrition beliefs did not uniquely explain variance in PIU, although both showed significant negative bivariate correlations with PIU (Table 3). This pattern suggests that their associations with PIU are largely shared with general health beliefs and the covariates rather than being domain-specific [40]. Prior research has shown that lifestyle-specific beliefs do not always translate into consistent behavioral patterns, particularly in populations facing high academic demands [10,41,42]. Among gifted children, beliefs related to physical activity and nutrition may be insufficient to independently relate to internet use behaviors in the absence of strong internalized health responsibility [22,40].

### 4.3. Health Literacy Domains and Problematic Internet Use

The health literacy domains did not add statistically significant incremental variance beyond the covariates and lifestyle beliefs (ΔR^2^ = 0.028, *p* = 0.495), and no individual domain was a statistically significant predictor in the adjusted model. These results should not be interpreted as demonstrating the absence of any association: self-awareness, citizenship, and practical knowledge showed significant negative bivariate correlations with PIU, and the possibility of a Type II error cannot be excluded given the modest sample size relative to the number of predictors and the psychometric fragility of the two-item domain scores (α = 0.47–0.63), which attenuates observed associations. Although health literacy is widely recognized as a determinant of health-related decision-making [10,11,12,13,14], the present findings indicate that cognitive health competencies alone may not explain problematic internet behaviors in this population. One plausible—but post hoc and untested—hypothesis for future research is that gifted children’s advanced information-processing skills and broad access to health information may reduce variability in health literacy scores, limiting statistical discriminative power [25,32]; the present study did not directly assess information-processing skills, and this interpretation should therefore be treated as hypothesis-generating rather than as an explanation supported by the current data. These findings underscore a critical distinction between possessing health knowledge and consistently acting on that knowledge in daily behavioral contexts [43,44].

### 4.4. Covariates and the Role of Screen Time

When sociodemographic and lifestyle covariates were controlled, daily screen time emerged as the strongest correlate of PIU across all steps. This is unsurprising given the conceptual proximity between time-based exposure and problematic use [33,34]; however, PIU is defined by loss of control and functional impairment rather than duration alone [1,3], and screen time and health beliefs contributed independently to the model in Step 2. The attenuation of the health beliefs coefficient in the full model (β = −0.21, *p* = 0.079) most plausibly reflects shared variance with the health literacy domains—particularly self-awareness (r = 0.48)—rather than the absence of an association; this pattern illustrates a general implication of the hierarchical strategy, namely that coefficients in the final step estimate unique contributions net of all other predictors and should be interpreted alongside the bivariate correlations and step-wise increments.

### 4.5. Implications for School Health Policy, Practice, and Equity

Within the constraints of cross-sectional evidence, these findings carry implications for school health nurses and other school health professionals. Interventions aimed at preventing PIU among gifted children may benefit from moving beyond information-based health education toward approaches that also address internalized health beliefs, self-regulation skills, and personal health responsibility. School health nurses are well positioned to implement counseling-based approaches that emphasize value clarification, goal setting, and the development of healthy digital routines tailored to the cognitive and emotional characteristics of gifted students; the effectiveness of such approaches in this population remains to be tested in intervention studies. From an equity perspective, gifted children enrolled in specialized educational settings represent a population whose distinct behavioral health needs are frequently overlooked in standard school health programs; the present findings highlight the importance of developing tailored, evidence-based interventions for this group.

### 4.6. Strengths and Limitations

This study has several strengths. It is, to the best of our knowledge, the first study to simultaneously examine health literacy domains and health-promoting lifestyle beliefs in relation to PIU specifically among gifted school-aged children. The use of validated instruments, the covariate-adjusted hierarchical analytical strategy, and the inclusion of a clearly defined, institutionally identified gifted sample strengthen the internal validity of the findings.

Several limitations should be considered. The cross-sectional design precludes causal inference; all reported associations are correlational, and reverse or bidirectional pathways (e.g., PIU eroding health beliefs) are equally compatible with the data. The convenience sample drawn from a single BİLSEM center in İzmir limits generalizability. Reliance on self-reported measures introduces the possibility of response bias. The two-item HLSAC domain scores showed modest internal consistency (α = 0.47–0.63), which may have attenuated their associations with PIU and increased the risk of Type II error in Step 3. The final thirteen-predictor model was specified during the revision process and exceeded the predictor set assumed in the a priori power analysis. The partial conceptual overlap between daily screen time (a covariate) and PIU may have produced conservative estimates for the belief and literacy predictors. The moderate explanatory power of the final model (R^2^ = 0.339) suggests that additional variables—such as parenting behaviors, peer influences, or emotional regulation capacities—likely contribute to PIU in this population. Future research using longitudinal designs and multi-center samples is needed.

## 5. Conclusions

Sociodemographic and lifestyle characteristics, health-promoting lifestyle beliefs—specifically health beliefs—contributed significantly to explaining PIU among gifted school-aged children, whereas health literacy domains did not add statistically significant incremental variance. Within the limits of a cross-sectional design, these findings suggest that school health nursing interventions addressing internalized health beliefs may usefully complement information-based education in supporting healthy digital engagement among gifted children.

## Figures and Tables

**Table 1:** Demographic and Lifestyle Characteristics of the Participants (N = 118).

Variable	Category	n (%)
Sex	Female	65 (55.1)
	Male	53 (44.9)
Chronic disease	None	101 (85.6)
	Allergic diseases	9 (7.6)
	Metabolic & endocrine disorders	2 (1.7)
	Cardiovascular & respiratory diseases	3 (2.5)
	Neurological & developmental disorders	1 (0.8)
	Rheumatologic & immune system diseases	1 (0.8)
	Other	1 (0.8)
Daily sleep duration	<6 h	15 (12.7)
	7–8 h	77 (65.3)
	9–10 h	23 (19.5)
	≥11 h	3 (2.5)
Daily screen time	<2 h	38 (32.2)
	3–4 h	54 (45.8)
	5–6 h	20 (16.9)
	>7 h	6 (5.1)
Weekly physical activity	None	34 (28.8)
	1–2 days/week	42 (35.6)
	3–4 days/week	22 (18.6)
	≥5 days/week	20 (16.9)

**Table 2:** Descriptive Statistics and Internal Consistency of the Study Measures (N = 118).

Measure	Mean ± SD	Observed Range	Cronbach’s α
PIUS-A total	61.58 ± 20.71	27–123	0.92
HLSAC total	31.81 ± 5.30	13–40	0.87
Theoretical knowledge	5.84 ± 1.33	2–8	0.61 ^a^
Practical knowledge	6.87 ± 1.20	2–8	0.56 ^a^
Critical thinking	6.14 ± 1.40	2–8	0.54 ^a^
Self-awareness	6.53 ± 1.36	2–8	0.63 ^a^
Citizenship	6.42 ± 1.21	3–8	0.47 ^a^
AHPLBS total	61.96 ± 11.85	24–80	0.91
Health beliefs	27.19 ± 6.21	9–35	0.87
Physical activity beliefs	20.61 ± 3.87	5–25	0.77
Nutrition beliefs	14.16 ± 3.71	6–20	0.85

^a^ Two-item domain; inter-item correlations were r = 0.31–0.46.

**Table 3:** Pearson Correlations Among PIU, Health-Literacy Domains, and Health-Promoting Lifestyle Beliefs (N = 118).

Variable	1	2	3	4	5	6	7	8
1. PIU total	—							
2. Theoretical knowledge	−0.16	—						
3. Practical knowledge	−0.21 *	0.57 **	—					
4. Critical thinking	−0.13	0.60 **	0.60 **	—				
5. Self-awareness	−0.27 **	0.51 **	0.64 **	0.60 **	—			
6. Citizenship	−0.23 *	0.57 **	0.53 **	0.56 **	0.64 **	—		
7. Health beliefs	−0.40 **	0.30 **	0.26 **	0.21 *	0.48 **	0.29 **	—	
8. Physical activity beliefs	−0.22 *	0.30 **	0.14	0.15	0.32 **	0.14	0.50 **	—
9. Nutrition beliefs	−0.38 **	0.37 **	0.26 **	0.34 **	0.53 **	0.31 **	0.68 **	0.62 **

* *p* < 0.05; ** *p* < 0.01 (two-tailed). Sociodemographic and lifestyle covariates were not included in this correlation matrix.

**Table 4:** Hierarchical Multiple Linear Regression Predicting Problematic Internet Use (N = 118).

Step/Predictor	B	SE	β	*p*	VIF
**Step 1**					
Age (years)	0.91	0.96	0.09	0.346	1.35
Male sex	3.57	3.44	0.09	0.303	1.03
Daily sleep duration	−4.21	2.92	−0.13	0.152	1.22
**Daily screen time**	**8.81**	**2.19**	**0.36**	**<0.001**	**1.16**
Weekly physical activity	−3.64	1.66	−0.19	0.031	1.06
**R^2^ = 0.248, adjusted R^2^ = 0.214, F(5,112) = 7.37, *p* < 0.001**					
**Step 2**					
Age (years)	0.49	0.96	0.05	0.609	1.43
Male sex	4.57	3.37	0.11	0.177	1.04
Daily sleep duration	−2.64	2.90	−0.08	0.363	1.28
Daily screen time	7.04	2.20	0.28	0.002	1.25
Weekly physical activity	−3.12	1.67	−0.16	0.064	1.13
Health beliefs	−0.79	0.37	−0.24	0.036	1.98
Physical activity beliefs	0.28	0.56	0.05	0.617	1.73
Nutrition beliefs	−0.51	0.71	−0.09	0.472	2.57
**R^2^ = 0.311, adjusted R^2^ = 0.260, ΔR^2^ = 0.063, F change(3,109) = 3.32, *p* = 0.023**					
**Step 3**					
Age (years)	0.63	0.98	0.06	0.520	1.50
Male sex	3.82	3.47	0.09	0.273	1.10
Daily sleep duration	−3.35	2.97	−0.10	0.262	1.34
Daily screen time	7.19	2.25	0.29	0.002	1.29
Weekly physical activity	−3.37	1.74	−0.17	0.055	1.22
Health beliefs	−0.69	0.39	−0.21	0.079	2.16
Physical activity beliefs	0.08	0.57	0.02	0.885	1.81
Nutrition beliefs	−0.56	0.77	−0.10	0.468	2.97
Theoretical knowledge	3.00	1.87	0.19	0.111	2.27
Practical knowledge	−2.23	2.05	−0.13	0.278	2.22
Critical thinking	0.54	1.76	0.04	0.761	2.22
Self-awareness	0.78	2.11	0.05	0.713	3.02
Citizenship	−2.73	1.99	−0.16	0.173	2.12
**R^2^ = 0.339, adjusted R^2^ = 0.256, ΔR^2^ = 0.028, F change(5,104) = 0.88, *p* = 0.495; Durbin–Watson = 2.05**					

Note: Age was entered in years, and sex was coded 0 = female and 1 = male. Daily sleep duration, daily screen time, and weekly physical activity were entered as ordinal scores from 1 to 4 in the order of the categories shown in Table 1. For these ordinal covariates, B represents the expected change in the PIU total score for a one-category increase.

## Data Availability

The data presented in this study are available on request from the corresponding author due to privacy and ethical restrictions.
